# Etiological analysis of graft dysfunction following living kidney transplantation: a report of 366 biopsies

**DOI:** 10.1080/0886022X.2018.1455592

**Published:** 2018-04-05

**Authors:** Jin Zhang, Jiang Qiu, Guo-Dong Chen, Chang-Xi Wang, Chang Wang, Shuang-Jin Yu, Li-Zhong Chen

**Affiliations:** Department of Organ Transplant, The First Affiliated Hospital of Sun Yat-sen University, Guangzhou, China

**Keywords:** Living kidney transplantation, graft dysfunction, biopsy, IgA nephropathy, rejection

## Abstract

**Aim:** The aim of this study is to investigate the clinical features of graft dysfunction following living kidney transplantation and to assess its causes.

**Methods:** We retrospectively analyzed a series of 366 living kidney transplantation indication biopsies with a clear etiology and diagnosis from July 2003 to June 2016 at our center. The classifications and diagnoses were performed based on clinical and pathological characteristics. All biopsies were evaluated according to the Banff 2007 schema.

**Results:** Acute rejection (AR) occurred in 85 cases (22.0%), chronic rejection (CR) in 62 cases (16.1%), borderline rejection (BR) in 12 cases (3.1%), calcineurin inhibitor (CNI) toxicity damage in 41 cases (10.6%), BK virus-associated nephropathy (BKVAN) in 43 cases (11.1%), de novo or recurrent renal diseases in 134 cases (34.7%), and other causes in nine cases (2.3%); additionally, 20 cases had two simultaneous causes. The 80 cases with IgA nephropathy (IgAN) had the highest incidence (59.7%) of de novo or recurrent renal diseases. After a mean ± SD follow up of 3.7 ± 2.3 years, the 5-year graft cumulative survival rates of AR, CR, CNI toxicity, BKVAN, and de novo or recurrent renal diseases were 60.1%, 31.2%, 66.6%, 66.9%, and 67.1%, respectively.

**Conclusions:** A biopsy is helpful for the diagnosis of graft dysfunction. De novo or recurrent renal disease, represented by IgAN, is a major cause of graft dysfunction following living kidney transplantation.

## Introduction

Biopsy is the gold standard for the diagnosis of graft dysfunction. Biopsy results change the clinical diagnosis in 36% and the therapy for 59% of patients [1]. An indication biopsy is used to assess the causes of graft dysfunction, which include rejection, *de novo* or recurrent renal diseases, infectious diseases, and drug toxicity, to provide guidance for treatment. Many pathological morphological studies have focused on graft dysfunction based on an indication biopsy, but few etiological analyses have been conducted in a large case series.

## Methods

### Sample collection

**Figure 1.**
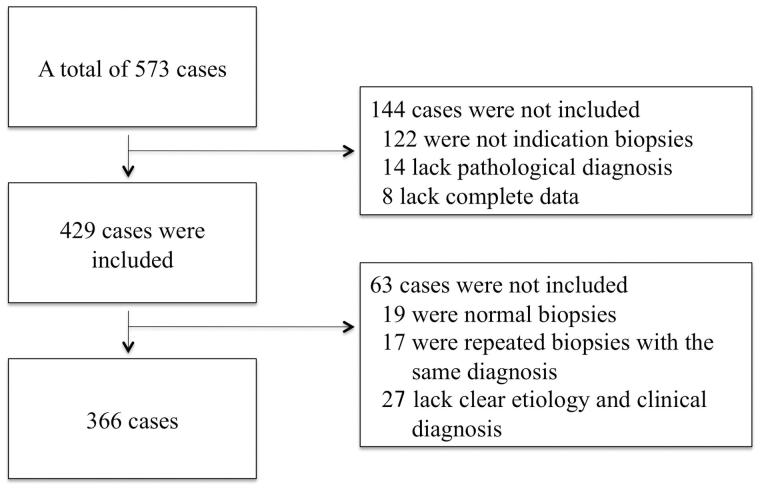
To investigate the causes of graft dysfunction, this series included patients with repeated indication biopsies, but excluded protocol biopsies and repeated indication biopsies with the same diagnosis. Additionally, biopsy results without a clear etiology and clinical diagnosis were excluded, such as chronic changes without evidence of any specific etiology (category 5 or 6 in Banff 2007).

A total of 366 patients who underwent an indication biopsy following living kidney transplantation from July 2003 to June 2016 at our center were included (Figure 1). The patients included 260 males and 106 females with a mean age (mean ± SD) of 38.6 ± 11.3 years (Table 1). Blood and urine routine examinations, liver and kidney function tests, and therapeutic drug monitoring were conducted before the biopsy for all patients. Induction treatment consisted of basiliximab (85.2%) or rabbit anti-thymocyte globulin (ATG) (14.8%); the standard maintain immunosuppressive regimen included a calcineurin inhibitor (CNI), FK506 (89%) or Cyclosporin A (CsA) (11%), combined with mycophenolate mofetil (MMF) (100%) and corticosteroids (100%); prednisone oral began with 30 mg, qd and withdrawal gradually to 5–10 mg, qd for a period of 4 weeks. This study was approved by the Regional Ethics Committee of our center, and all patients signed informed consent forms.

**Table 1.** Recipient and donor characteristics.

Variable	
Recipient age (years)	38.6 ± 11.3 (7–68)
Recipient sex, f/m (%)	106/260 (29/71)
Recipient race, East Asian (%)	366 (100)
Time on dialysis before Tx (months)	25.7 ± 15.3 (0–52)
Diabetes (%)	71 (19.4)
Hypertension (%)	332 (90.7)
Second or higher Tx (%)	12 (3.2)
Anti-HLA antibodies^a^ (%)	76 (20.8)
Class I (%)	29 (7.9)
Class II (%)	54 (14.7)
HLA mismatches (A, B, or DR)	2.2 ± 1.3
Donor age (years)	51.2 ± 8.4 (36–65)
Donor sex f/m (%)	218/148 (59/41)
Donor race, East Asian (%)	366 (100)
Related donors (%)	318 (87)
Follow-up (years)	3.7 ± 2.3

Tx: transplantation.

a Including donor-specific antibody and non-donor specific antibody.

### Indication and procedures in biopsy

The indications for the biopsy with reference to 2009 KDIGO Clinical Practice Guideline [2] were as follows: (1) continuous anuria or oliguria; (2) durative hematuria or proteinuria; (3) continuous increase in the serum creatinine (sCr) or a concentration above the normal level; (4) B-scan ultrasonography showing an abnormal blood flow resistance index (RI); (5) panel-reactive antibody (PRA) > 0% or presence of a donor-specific antibody (DSA).

A needle biopsy guided by ultrasonography was performed with an 18-gauge needle (Bard). Each sample had at least six glomeruli under light microscopy, electron microscopy, and immunofluorescence analyses (for IgA, IgG, IgM, C1q, and C4D) were performed on all biopsies and were observed by two senior pathologists in an independent and blinded fashion. The diagnosis and classification were determined by senior pathologist, some were consulted with doctor in charge, according to the Banff 2007 schema combined with clinical examinations, including therapeutic drug monitoring and BK virus testing in the urine and sera.

### Statistical analysis

We analyzed the incidence and the 5-year graft cumulative survival rate of the various causes of graft dysfunction. Due to a great discrepancy in diseased time of different causes, the survival time after transplantation may not reflect the clinical features of graft dysfunction, we defined the time of follow-up began from the day of diagnosis by biopsy instead of the day of transplantation.

Data were analyzed with IBM SPSS Statistics software version 22.0 (SPSS, Chicago, IL). The comparisons of categorical variables were performed with the Mann–Whitney *U* test or chi-square test, *p* values lower than .05 were considered statistically significant, the multiple comparisons were performed with Kruskal–Wallis test and the results were corrected with Bonferroni correction.

## Results

**Figure 2.**
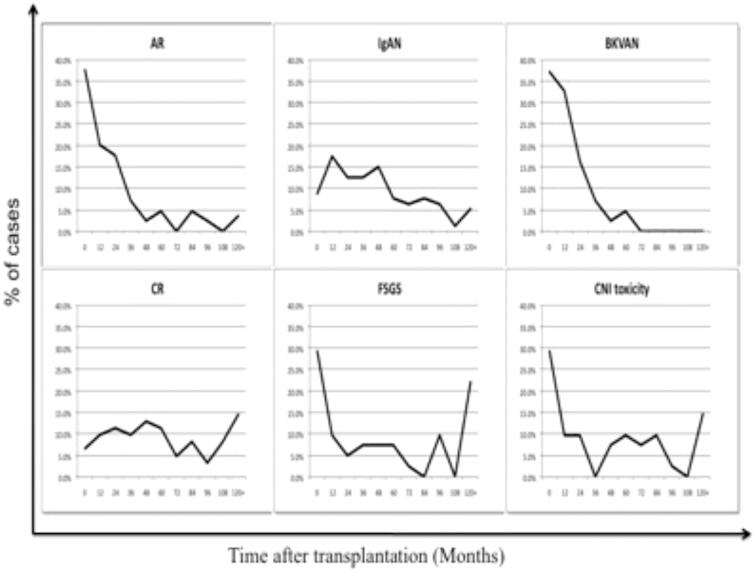
The incidence of various causes in different clinical settings. AR was established within the first year after transplantation and then declined sharply in the following years. The peak incidence of BKVAN occurred within 1–2 years postoperatively. FSGS occurred more frequently within the first year and nearly 10 years postoperatively. IgAN, CR, and CNI toxicity damage occurred similarly in each year.

The 366 cases (Table 2) included 85 cases (22.0%) of acute rejection (AR), the glomerulitis (g > 0) was found in 52 cases (61.2%), and the peritubular capillaritis (ptc >0) in 73 cases (85.9%); of which 14 cases were acute antibody-mediated rejection (AAMR), all these biopsies (16.5%) had ptc and were C4d-positive by immunohistochemical staining. Thirty-two cases occurred within the first year after transplantation; nevertheless, three cases occurred more than 10 years postoperatively (Figure 2). Patients with T cell-mediated rejection (ACMR) were treated with a methylprednisolone pulse (MPP) and ATG intravenous therapy. Additionally, anti-CD20 monoclonal antibody intravenous immunoglobulin (IVIG) therapy was added to patients considered as antibody-mediated. Of these patients, 84.7% recovered and 15.3% lost graft within 1 year after diagnosis. Overall, the 5-year graft cumulative survival rate of AR was 60.1%.

**Table 2.** The incidence of various causes of graft dysfunction.

Causes of graft dysfunction	Count (*n* = 386^a^, %)
De novo or recurrent renal diseases	134 (34.7)
IgAN	80 (59.7)
FSGS	41 (30.6)
MN	6 (4.5)
MPGN	4 (3.0)
LPG	2 (1.5)
LN	1 (0.7)
AR	85 (22.0)
CR	62 (16.1)
BKVAN	43 (11.1)
CNI toxicity	41 (10.6)
BR	12 (3.1)
Others	9 (2.3)

a This count is the incidence of causes of graft dysfunction in 366 cases, including two causes occurred in one case simultaneously.

Chronic rejection (CR) occurred in 62 cases (16.1%), and the occurrence was similar in each year, as well as 41 cases (10.6%) of CNI toxicity damage. We adjusted the dosage according to the blood concentrations or converted immunosuppression from CNI to rapamycin (RAPA). With early control of the blood pressure and hyperlipidemia, the 5-year graft cumulative survival rates were 31.2% and 66.6%, respectively.

Forty-three cases (11.1%) had BK virus-associated nephropathy (BKVAN), most of which occurred within 1–2 years after transplantation. None of the patients were diagnosed with BKVAN after more than 7 years. After the treatment of immunosuppression reduction and replacement of FK506 with CsA, the 5-year graft cumulative survival rate was 74.3%.

De novo or recurrent renal diseases occurred in 134 cases, representing the highest incidence (34.7%) (Table 2). These diseases included IgA nephropathy (IgAN) in 80 cases (59.7%), focal segmental glomerulosclerosis (FSGS) in 41 cases (30.6%), membranous nephropathy (MN) in six cases (4.5%), membranoproliferative glomerulonephritis (MPGN) in four cases (3.0%), lipoprotein glomerulopathy (LPG) in two cases (1.5%), and lupus nephritis (LN) in one case (0.7%). IgAN and FSGS were predominant. These two diseases occurred at any time after transplantation, although the latter disease occurred more frequently within the first year and nearly 10 years postoperatively. CsA and medium/low prednisone combination therapy was used to relieve disease progression, angiotensin-converting enzyme inhibitors (ACEIs) were used to reduce proteinuria, and patients with FSGS were treated with plasma exchange (PE). As a result, the 5-year graft cumulative survival rates of these two diseases were 67.7% and 54.5%, respectively. For comparison, the 5-year graft cumulative survival rate for the total 134 cases was 67.1%.

Graft dysfunction with other causes occurred in nine cases (2.3%), including acute pyelonephritis, hypertension, and chronic obstruction nephropathy. Twenty cases had two causes simultaneously; these causes were AR and CR in four cases, *de novo* or recurrent renal disease and CNI toxicity damage in five cases, *de novo* or recurrent renal disease and CR in five cases, and *de novo* or recurrent renal disease and AR in six cases.

## Discussion

With the wide use of effective immunosuppression, the incidence of AR has been reduced markedly in recent years [3]. The overall risk within one year after transplantation is less than 15% [4]. In our study, the incidence of AR was second to the risk of *de novo* or recurrent renal diseases. However, the reduction in the incidence was not associated with a better prognosis, and the rejection episodes were more severe than previously reported [5]. Due to improved immunosuppression and management during the perioperative period, some cases of AR, especially those in subclinical rejection, showed fewer typical clinical features, such as an acute increase in creatinine with oliguria, fever and graft tenderness and swelling. Thus, renal biopsy is one of the main methods to predict and guide treatment.

AR has a high incidence in the first year but can occur at any time after transplantation [4]. This pattern was also observed in our study, with some cases occurring more than 10 years postoperatively. This result suggests that late AR must be noted and may occur when immunosuppression is reduced due to cancer, infection, or drug toxicity. Late AR, which is defined as a rejection occurs more than 3 months post-transplantation. Humoral factors have been reported to take effect in late AR and AAMR is observed in more than 40% of late AR cases [6,7]. AAMR is one of the most common and severe causes of graft dysfunction, and is characterized by hormone resistance and refractory. One study found that AAMR occurred in nearly 24% of the AR biopsies [8]. Fourteen biopsies (16.5%) showed AAMR in our study. Considering the low risk of rejection in living transplantation, this result was coincident with reports in the literature.

Although most AR cases can be cured, many studies have indicated that AR is still the main reason cause of graft dysfunction [9,10] and accounts for one-third of graft losses [11,12]. Late AR is often associated with poorer outcomes and a high risk of graft loss. We found that the 29 cases (96.6%) of AR that occurred within 2 year after transplantation (Group 1) recovered with anti-rejection treatment compared with the curative effective rate of only 86.0% in the 50 cases that occurred over one year postoperatively (Group 2) (Table 3), although there is no statistically significant in cure rate between two groups, the treatment outcome of group 1 is better than group 2 as can be seen from the decline degree of sCr. Moreover, the biopsies showed a higher degree of histopathological injury in Group 2 versus in Group 1. A meta-analysis showed that early steroid avoidance or withdrawal after renal transplantation increased the risk of late AR [13]. Therefore, the benefits and risks of immunosuppression reduction should be further assessed.

**Table 3.** The clinical features and treatment outcomes of early and late AR.

	Group 1 (*n* = 30)^a^	Group 2 (*n* = 50)^a^	*p* value
Recipient age (years)	35.8 ± 14.3 (10–68)	37.0 ± 10.6 (9–59)	.70
Anti-HLA antibodies (%)	14 (17.5)	31 (38.7)	.18
Class I (%)	7 (8.7)	13 (16.2)	
Class II (%)	9 (11.2)	24 (30.0)	
HLA mismatches (A, B, or DR)	2.6 ± 1.4	2.4 ± 1.2	.54
Induction treatment			.83
Basiliximab (%)	24 (30.0)	39 (48.7)	
ATG (%)	6 (7.5)	11 (13.7)	
CNI			.33
FK506 (%)	27 (33.7)	41 (51.2)	
CsA (%)	3 (3.7)	9 (18)	
AAMR (%)	8 (10.0)	6 (7.5)	.09
g^b^ (%)	13 (43.3)	35 (70.0)	.02
ptc (%)	25 (83.3)	43 (86.0)	.75
sCr baseline^c^ (umol/L)	126.0 ± 55.1	124.6 ± 53.2	.87
sCr increased^d^ (umol/L)	157.8 ± 244.9	169.0 ± 247.8	.55
sCr decline^e^ (umol/L)	69.5 ± 77.3	39.4 ± .2	.04
Cured^f^ (%)	29 (96.6)	43 (86.0)	.12

ATG: rabbit anti-thymocyte globulin; CNI: calcineurin inhibitor; CsA: Cyclosporin A; AAMR: acute antibody-mediated rejection; g: glomerulitis; ptc: peritubular capillaritis; sCr: serum creatinine.

a Five cases of AR were lost to follow up, three cases in group 1, and two cases in group 2 lost graft and returned to dialysis after biopsy without anti-rejection treatment.

b The g was defined as glomerulitis >0 according to the Banff 2007 schema, the same as the ptc.

c The sCr baseline was the lowest sCr level after transplantation.

d The increased from the sCr baseline to the sCr level at the time of biopsy.

e The decline from the sCr level at the time of biopsy to the level one week after anti-rejection treatment.

f The criterions of cure included clinical symptoms disappeared, the sCr and RI in B-scan ultrasonography recovered.

Unlike in deceased donors, in living transplantation, early steroid withdrawal has been shown to be a safe intervention in the management of low immunological risk [14,15]. However, some studies have suggested that the risk of recurrence has increased as a result [16,17]. This finding could help explain why *de novo* or recurrent renal diseases occupied the first position of the various causes of graft dysfunction in our results.

All forms of original nephropathy may recur after transplantation, including IgAN, FSGS, MN, and MPGN. However, great discrepancies exist in their clinical features and prognoses. Data have shown that *de novo* or recurrent renal diseases are the third leading cause of graft dysfunction, following CR and death with functional graft [18].

IgAN is the most common recurrent nephropathy and recurs in 9–61% of patients [19]. Moreover, IgAN had the highest incidence in our study. Genetic factors play a role in the recurrence of IgAN and exhibit familial clustering [20]. We noted that IgAN recurrence was significantly more frequent in low HLA-mismatched living transplantation, which conformed to a review of the Australia–New Zealand registry [21]. Therefore, we suggest that further attention should be paid to the family history in cases involving transplantation from living donors. IgAN may recur at any time after transplantation. We found that most patients diagnosed with recurrent IgAN accepted a biopsy for initial asymptomatic hematuria and/or mild proteinuria, whereas few for an increased serum creatinine level, which was contrary to the rejection and CNI toxicity data (Table 4). Early research suggested that recurrent IgAN presented relatively slow progression and a benign outcome with little impact on graft function compared with other risk factors, such as rejection, drug toxicity, or infection [22]. More recent studies have confirmed that IgAN reduces the long-term survival rate of the graft and indicates a poor prognosis, especially in patients with asymptomatic hematuria and/or proteinuria [23,24].

**Table 4.** The clinical features of different causes at the time of biopsy.

	IgAN (*n* = 80)	AR (*n* = 85)	CR (*n* = 62)	CNI toxicity (*n* = 41)	*p* value
Proteinuria (−∼+++)	80	85^a^	62	41^a^	<.01
− (%)	20 (25.0)	52 (61.2)	19 (30.6)	24 (58.5)	
+ (%)	31 (38.7)	14 (16.5)	18 (29.0)	12 (29.3)	
++ (%)	20 (25.0)	8 (9.4)	14 (22.6)	3 (7.3)	
+++ (%)	9 (11.2)	11 (12.9)	11 (17.7)	2 (4.9)	
24-h urinary protein (g/24 h)	1.14 ± 1.47	0.82 ± 0.93	1.80 ± 2.55	0.69 ± 0.78	.05
Haematuria (−∼+++)	80	85^a^	62^a^	41^a^	<.01
− (%)	24 (30.0)	54 (63.5)	36 (58.1)	28 (68.3)	
+ (%)	26 (32.5)	17 (20.0)	20 (32.2)	8 (19.5)	
++ (%)	21 (26.2)	7 (8.2)	4 (6.4)	4 (9.7)	
+++ (%)	9 (11.2)	7 (8.2)	2 (3.2)	1 (2.4)	
Urinary RBC count (µ/ml)	64.1 ± 95.7	111.2 ± 413.1^a^	36.8 ± 132.9^a^	51.7 ± 239.3^a^	<.01
Cholesterol (mmol/L)	5.5 ± 1.9	5.4 ± 2.0	5.6 ± 1.3	5.3 ± 1.3	.23
LDL (mmol/L)	3.6 ± 1.4	3.6 ± 1.5	3.9 ± 1.1	3.5 ± 1.0	.21
ALB (g/L)	40.7 ± 5.9	41.5 ± 5.3	38.8 ± 5.7	42.6 ± 4.1	<.01
sCr increased (µmol/L)	42.1 ± 69.2	160.6 ± 238.5^a^	121.8 ± 138.8^a^	61.4 ± 76.7	<.01
Diseased time (years)	4.0 ± 3.1	2.2 ± 2.6^a^	5.4 ± 4.0	4.3 ± 4.0	<.01

RBC: red blood cell; LDL: low density lipoprotein; ALB: albumin.

a The significant difference between the IgAN group and the other three groups, *p* values lower than .008 (0.05/6) were considered statistically significant with Bonferroni correction.

Recurrent FSGS develops in approximately 30% of patients after the first transplantation [25]. We found that this disease occurred more frequently within the first year and nearly 10 years postoperatively. Early cases were characterized by severe proteinuria within hours to days after transplantation. In contrast, the late cases developed asymptomatic proteinuria within several months to years. In some cases, the primary diseases of the recipients were unknown; therefore, distinguishing between recurrent FSGS and *de novo* cases is difficult. Thus, we conjecture that these two periods may represent a peak incidence of recurrent and *de novo* FSGS, respectively. To test the hypothesis, more biopsy samples should be collected preoperatively. Recurrent FSGS is prone to occur in males and children [26], but most patients in our study were male (71.4%) and adults (100%). Although few studies have reported data on prognoses in adult cases with recurrent FSGS, we can acknowledge that recurrence is associated with poor graft survival. Moroni et al. [26] reported recurrent FSGS in 12 adult patients; graft failure occurred in seven of the patients within 10 months, and five patients retained good functions at 106 months. Our study uncovered a similar result, with a 5-year graft cumulative survival rate of 54.5%.

BKVAN resulted in graft failure in 7% of the cases and emerged as one of the most important infectious diseases after transplantation [11]. Significant BK viruria and viremia is common following transplantation within the first year and subsequently proceeds to BKVAN. Therefore, we observed a peak incidence of BKVAN diagnosed by biopsy within 1–2 years postoperatively in our study. Although no patient was diagnosed with BKVAN after more than 7 years, further research is needed to determine whether BKV infections can influence the long-term prognosis. Immunosuppressant reduction treatment and replacement of FK506 with CsA are effective in BKV infection recipients at an early stage. Our previous study provided successful resolution of BK viremia and BKVAN with excellent graft survival at 5 years by intensive monitoring and pre-emptive immunosuppression reduction [27]. Recently, one study called for the use of pre-transplantation BK viral serological testing of living donors and recipients, which provided a serological marker that predicted the risk of BK viremia and BKVAN after transplantation [28]. Subsequent studies are needed to increase the efficiency of BK virus screening.

Since the pathological and immunological mechanisms remain unknown, no reliable treatment strategy has been proven to reverse the progression of some diseases, such as CR, recurrent IgAN, and FSGS. However, biopsy promotes the detection rate of the causes of graft dysfunction. Early and aggressive treatment could be provided to improve the prognosis.

## Conclusions

A biopsy is helpful for the diagnosis of graft dysfunction. To this end, we conclude that *de novo* or recurrent renal disease, represented by IgAN, is more frequent than rejection as a major cause of graft dysfunction following living kidney transplantation. When choosing to maintain immunosuppressive treatments for living transplantation, especially in patients with a high risk of recurrence, steroid withdrawal should be handled with caution, and an early biopsy should be performed.

## Limitation

Despite the incidence and 5-year graft cumulative survival rates analyzed in our study, we did not perform a comparison of the significance between groups for the prognosis of graft dysfunction, influenced by the lack of information about the primary diseases of the recipients and other donor factors. In addition, a few patients did not visit and have biopsy in time when having an initial indication. So we could not compare the outcomes of the different causes in this study. To achieve this end, a complete database and survival analysis based on Cox regression is needed in a subsequent study.
